# Comparison of direct anterior vs. posterior approach in primary total hip arthroplasty: a systematic review and meta-analysis on enhanced recovery after surgery

**DOI:** 10.3389/fsurg.2025.1586187

**Published:** 2025-11-06

**Authors:** Wenqian Xu, Jinjin Lao, Jinsong Liu, Zengrui Zhang, Xiaoyu Wan, Zhiguang Chen, Xiaotao Huang, Nan Chen, Yingxing Xu

**Affiliations:** 1 Department of Orthopaedics, First Affiliated Hospital of Kunming Medical University, Kunming, Yunnan, China; 2 Department of Orthopaedics and Traumatology, Cixi Hospital of Traditional Chinese Medicine, Ningbo, Zhejiang, China; 3 Department of Trauma Center, First Affiliated Hospital of Kunming Medical University, Kunming, Yunnan, China

**Keywords:** direct anterior approach, posterior approach, total hip arthroplasty, enhanced recovery after surgery, meta-analysis

## Abstract

**Purpose:**

This meta-analysis aimed to compare the direct anterior approach (DAA) and posterior approach (PA) for total hip arthroplasty (THA) within the context of enhanced recovery after surgery (ERAS).

**Methods:**

Studies comparing DAA and PA for THA were systematically retrieved from PubMed, Embase, Web of Science, Cochrane Library, and Google Scholar databases, covering the period from 2012 to 2024. A meta-analysis was conducted to compare the ERAS-related outcomes between DAA and PA for THA using RevMan 5.3 software, including surgical trauma, muscle damage, functional recovery, and complications. Heterogeneity was considered significant if *I*^2^ > 50%, in which case a random-effects model and subgroup analysis were applied. Continuous and dichotomous data were analyzed using 95% confidence intervals (CIs). Methodological quality and heterogeneity assessments were also conducted.

**Results:**

A total of 48 studies, including 46,367 hips (13,285 in the DAA group and 33,082 in the PA group), were included. Compared with PA, DAA was associated with significantly lower blood transfusion rates [6.62% vs. 14.52%; odds ratio (OR) = 0.73; 95% CI: 0.59–0.91; *P*  < 0.005], shorter hospital stay [mean difference (MD) = −0.88 days; 95% CI: −1.10 to −0.87; *P* < 0.001], and less gluteus minimus muscle damage on magnetic resonance imaging (MRI) (36.84% vs. 65.79%; OR = 0.28; 95% CI: 0.14–0.56; *P* < 0.005). Lower levels of creatine kinase (MD = −49.58; 95% CI: −56.43 to −43.26; *P* < 0.001) and C-reactive protein (MD = −4.48; 95% CI: −5.28 to −4.47; *P* < 0.001) were also observed in the DAA group. Functional outcomes, including Harris hip score (MD = 3.07; 95% CI: 0.08–6.07; *P* < 0.05) and short form (SF) score (MD = 1.53; 95% CI: 0.80–2.26; *P* < 0.001), were better with DAA. Dislocation rates were significantly lower with DAA (0.84% vs. 1.82%; OR = 0.32; 95% CI: 0.21–0.48; *P* < 0.001). However, there were no significant differences between DAA and PA in surgery time (MD = 2.43; 95% CI: −2.20 to 7.06; *P* = 0.30), gluteus medius muscle damage on MRI (17.34% vs. 15.15%; OR = 1.20; 95% CI: 0.53–2.71; *P* = 0.66), tensor fasciae latae muscle damage on MRI (25.51% vs. 38.38%; OR = 0.40; 95% CI: 0.03–4.97; *P* = 0.48), time to discontinuation of assistive devices (MD = −1.85; 95% CI: −4.05 to 0.35; *P* = 0.10), infection (1.09% vs. 0.60%; OR = 0.92; 95% CI: 0.48–1.77; *P* = 0.81), nerve injury (0.60% vs. 0.68%; OR = 1.06; 95% CI: 0.69–1.64; *P* = 0.79), intraoperative fracture (0.55% vs. 0.79%; OR = 0.68; 95% CI: 0.36–1.26; *P* = 0.22), or leg length discrepancy (MD = −1.85; 95% CI: −4.05 to 0.35; *P* = 0.10).

**Conclusion:**

Within the framework of ERAS, the DAA was found to be associated with reduced muscle damage, fewer postoperative complications, and improved functional recovery compared with the PA in patients undergoing THA.

**Systematic Review Registration:**

https://www.crd.york.ac.uk/PROSPERO/recorddashboard.

## Introduction

Total hip arthroplasty (THA) is one of the most effective treatments for end-stage hip disorders (1). Various surgical approaches have been developed for THA, including the posterior approach (PA), direct anterior approach (DAA), lateral approach, and minimally invasive techniques such as the orthopaedic chirurgie München (OCM) and the supercapsular percutaneously assisted total hip (SUPER-PATH) approach (2–4). Among these, the DAA has gained widespread clinical adoption as a representative minimally invasive technique. The approach was performed through the muscle gap between the broad fascia tensor and the sartorius muscle, allowing for muscle-sparing access to the hip joint (5). Compared with the PA, the DAA has been associated with several benefits from enhanced recovery after surgery (ERAS), including reduced muscle damage, faster postoperative recovery, and less pain (6). Although numerous studies have compared the DAA and PA in terms of complications, surgery time, length of hospital stay, muscle damage, and functional outcomes (7–13), few have systematically evaluated these parameters within the framework of ERAS. A comprehensive comparison of ERAS-related outcomes between these two approaches is essential to inform surgical decision-making and to identify which technique aligns more closely with ERAS principles. Therefore, we conducted a meta-analysis to compare the DAA and PA approaches in the context of ERAS. Key outcomes included surgical trauma (blood transfusion rate, hospital stay, and surgery time), muscle damage [magnetic resonance imaging (MRI) and serum creatine kinase (CK) and C-reactive protein (CRP) levels], functional recovery [Harris hip score (HHS), short form (SF) score], and postoperative complications (dislocation rate, nerve injury rate, intraoperative fracture, infection rate, and leg length discrepancy).

## Materials and methods

This study was performed according to the Preferred Reporting Items for Systematic Reviews and Meta-Analyses (PRISMA) guidelines (14). Details of the protocol for this systematic review were registered on PROSPERO (CRD42051054229).

### Literature search

A comprehensive literature search was performed across English-language databases, including PubMed, Embase, Web of Science, Cochrane Library, and Google Scholar, as well as Chinese-language databases, including the China National Knowledge Infrastructure (CNKI), WanFang, and VIP, covering the period from 2012 to 2024. The search terms used were as follows: (“direct anterior approach” OR “DAA” OR “Hueter approach” OR “SmithPetersen approach”) AND (“posterior approach” OR “posterior lateral approach” OR “Kocher approach” OR “Gibson approach” OR “PA” OR “posterolateral approach”) AND (“total hip arthroplasty” OR “total hip replacement” OR “THA”). In addition, relevant articles cited in the reference lists of systematic reviews or meta-analyses were screened and included if they met the eligibility criteria.

### Inclusion criteria

Studies were included if they met the following criteria:

1. comparative studies evaluating DAA vs. PA or posterolateral approach in THA;
2. study design of randomized controlled trials (RCTs), prospective cohort studies, or retrospective studies; and
3. studies reported at least one of the following outcomes: hospital stay, surgery time, blood transfusion rate, MRI, CK level, CRP level, HHS, time to discontinuation of assistive devices, SF score, or postoperative complications;

### Exclusion criteria

Studies were excluded if they met any of the following conditions:

1. studies involving revision of THA;
2. study design of case reports, systematic reviews, meta-analyses, letters to the editor, and fundamental research;
3. studies including the assistance of computer navigation- and robot-assisted THA, or hemiarthroplasty; and
4. studies containing incomplete or unavailable data;

### Data extraction

Standardized data extraction forms were developed to collect the following information: (1) first author's surname; (2) year of publication; (3) methodological characteristics; (4) clinical data, including sample size, age range, and gender ratio; (5) follow-up duration; and (6) ERAS-related indicators, including blood transfusion rate, hospital stay, surgery time, MRI findings, CK and CRP levels, HHS, SF score, dislocation rate, infection rate, nerve injury rate, intraoperative fracture, and postoperative leg length discrepancy. Two reviewers (WX and JLa) independently extracted the data based on these forms. Any disagreements were resolved through consultation with a senior investigator (YX).

### Assessment of risk of bias

The risk of bias (ROB) for the included studies was assessed using the Newcastle–Ottawa scale (NOS) (15), methodological index for non-randomized studies (MINORS) (16) for non-randomized studies, and the Cochrane Collaboration's Risk of Bias tool for RCTs (17). Two reviewers (WX and JLa) independently conducted the assessments. Any disagreements were resolved through consultation with a senior investigator (YX).

### Statistical analysis

Statistical analyses were performed using Review Manager (RevMan) version 5.3 (Cochrane Collaboration, Oxford, UK). Odds ratios (ORs) were used for dichotomous outcomes, while weighted MDs were used for continuous variables. *P* < 0.05 was considered statistically significant. Heterogeneity among studies was assessed using the *I²* statistic derived from the chi-square test. An *I²* value of >50% indicated high heterogeneity, while an *I²* value of <50% suggested low heterogeneity. A fixed-effects model was applied when *P* > 0.1 and *I²* < 50%, whereas a random-effects model was used when *I²* exceeded 50%. When five or more studies were included, publication bias was assessed using Egger’s test with Stata software (version 17.0, StataCorp LP, College Station, TX, USA).

## Results

### Search results

**Figure 1:**
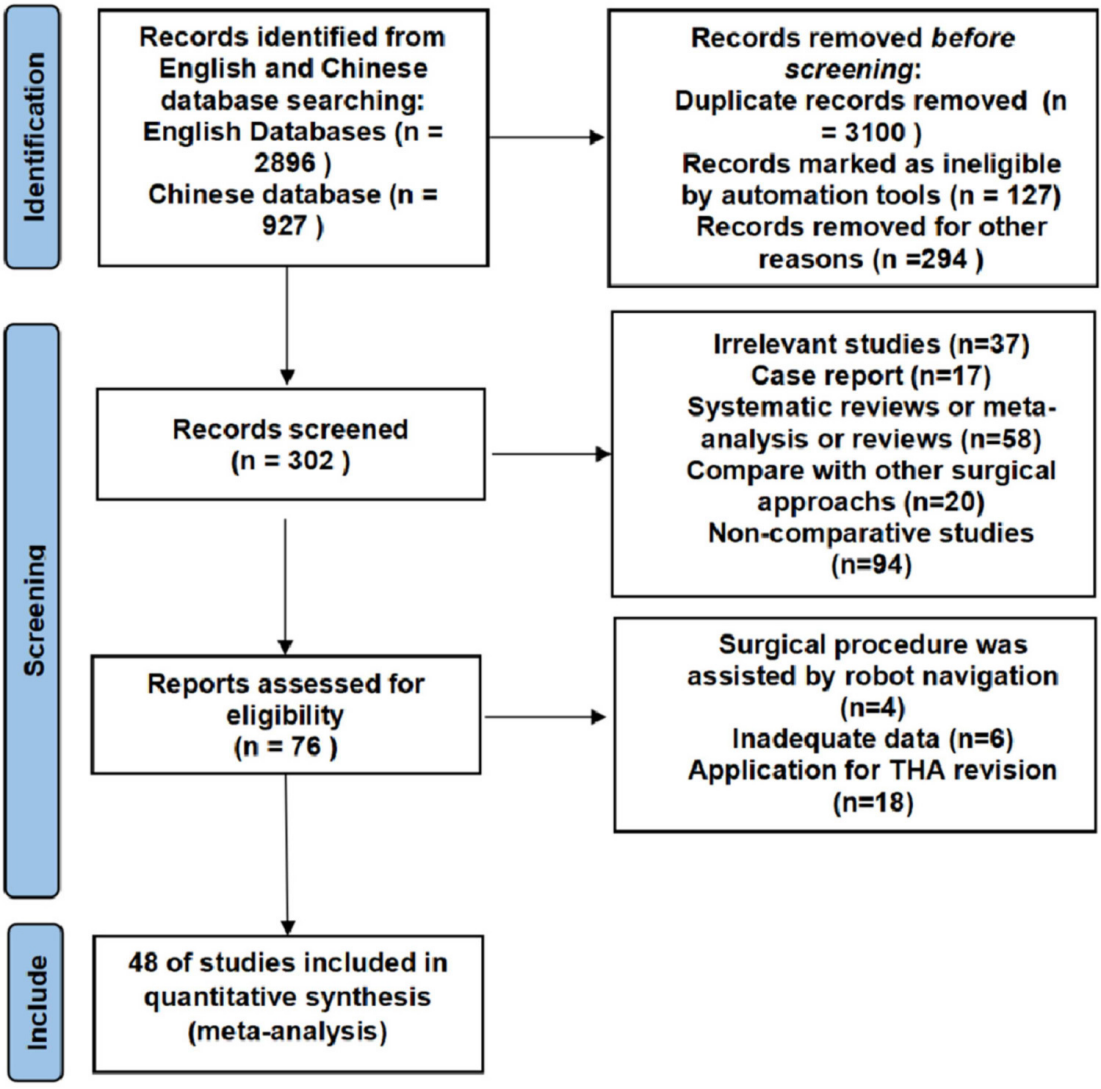
Meta-analysis flowchart.

A total of 3,823 studies were initially identified, of which 3,579 were excluded due to duplication or irrelevance. An additional 226 articles were excluded after screening the titles and abstracts. Following full-text review, 48 English-language articles that met the inclusion criteria were selected for meta-analysis. The detailed search and screening process is listed in Figure 1.

### Baseline characteristics of the included studies

A total of 48 articles were included in the meta-analysis: 4 RCTs and 44 case–control studies, of which 8 were prospective and 36 were retrospective. These studies included data on 46,367 hips, with 13,285 in the DAA group and 33,082 in the PA group. The included studies were published between 2012 and 2024, with a maximum follow-up duration of 4 years. Detailed baseline characteristics are provided in Table 1.

**Table 1:** Baseline characteristics of the included studies.

Study (authors, year)	Sample size	Study type	Age (years)	Gender (*n*)	BMI (mean ± SD/range)	Final follow-up time
			mean ± SD/range	(M/F)		
	(No. of hips)					
Di Martino et al., 2023 (18)	DAA (100)	Retrospective study	DAA (75)	DAA (42/58)	DAA (24)	DAA (15 months)
	PA (62)		PA (74)	PA (16/46)	PA (24)	
						PA (60 months)
Martusiewicz et al., 2020 (19)	DAA (55) PA (56)	Prospective study	DAA (63)	DAA (22/33)	DAA (29.3)	6 weeks
			PA (62)	PA (21/35)	PA (31.7)	
Spaans et al., 2012 (20)	DAA (46)	Retrospective study	DAA (69 ± 9.8)	DAA (24/22)	DAA (25 ± 3.0)	1 year
	PA (46)		PA (68 ± 11)	PA (14/32)	PA (29 ± 4.3)	
Bremer et al., 2011 (21)	DAA (25)	Retrospective study	DAA (70)	DAA (10/15)	DAA (26)	1 year
	PA (25)		PA (60)	PA (14/11)	PA (25)	
Fransen et al., 2016 (22)	DAA (45)	Retrospective study	DAA (25 ± 2.8)	DAA (15/30)	DAA (25 ± 2.8)	1 year
	PA (38)		PA (27.6 ± 3.2)	PA (13/22)	PA 27.6 ± 3.2)	
Zhang et al., 2022 (23)	DAA (127)	Retrospective study	DAA (52.00 ± 12.42)	DAA (94/33)	DAA (25.45 ± 3.47)	1 year
	PA (121)			PA (91/30)	PA (24.82 ± 3.71)	
			PA (51.15 ± 11.88)			
Chen et al., 2020 (24)	DAA (46)	Retrospective study	DAA (59.6 ± 6.0)	DAA (15/31)	DAA (22.72 ± 3.00)	1year
	PA (43)		PA (60.2 ± 5.0)	PA (14/29)	PA (21.67 ± 2.90)	
Yin et al., 2024 (25)	DAA (50)	Retrospective study	DAA (66.24 ± 4.15）	DAA (28/22)	DAA (64.58 ± 2.87)	1 year
	PA (44)		PA (66.28 ± 4.17)	PA (24/20)	PA (64.62 ± 2.91)	
Agten et al., 2017 (26)	DAA (30)	Retrospective study	DAA (63 ± 9)	DAA (10/20)	N/A	3 months
	PA (30)		PA (68 ± 12)	PA (11/19)		
Ponzio et al., 2018 (27)	DAA (289)	Retrospective study	DAA (65.1 ± 9.8)	DAA (122/167)	DAA (28.4 ± 5.5)	4 years
	PA (4,249)		PA (64.7 ± 11.2)		PA (28.1 ± 5.7)	
				PA (1,900/2,349)		
Maldonado et al., 2019 (28)	DAA (205)	Retrospective study	DAA (59.3 ± 8.8)	DAA (86/119)	DAA (29.4 ± 4.6)	2 years
	PA (205)		PA (58.8 ± 10.5)	PA (86/119)	PA (29.6 ± 4.7)	
Rhea et al., 2020 (29)	DAA (37)	Retrospective study	DAA（71）	DAA (16/21)	DAA (29.4)	DAA (44 months)
						PA (105 months）
	PA (37)		PA (66)	PA (16/21)	PA (29.5)	
Pala et al., 2016 (30)	DAA (55)	Retrospective study	DAA (89)	DAA (11/44)	N/A	6 months
	PA (54)		PA (87.6)	PA (10/44)		
Vasarhelyi et al., 2020 (31)	DAA (9)	Retrospective study	DAA (66.22 ± 8.53)	DAA (4/5)	DAA (27.02 ± 4.34)	1 year
	PA (7)		PA (64.14 ± 10.76)	PA (4/3)	PA (28.90 ± 5.86)	
Triantafyllopoulos et al., 2019 (32)	DAA (1,182)	Retrospective study	DAA (62.3 ± 10.8)	DAA (470/623)	N/A	71 months
			PA (64.2 ± 11.9)			
	PA (18,853)			PA (8,056/10,098)		
Sprowls et al., 2020 (33)	DAA (64)	Retrospective study	N/A	N/A	N/A	3 months
	PA (60)					
Zhao et al., 2017 (34)	DAA (60)	RCT	DAA (64.88 ± 12.13)	DAA (24/36)	DAA (24.35 ± 3.10)	6 months
	PA (60)			PA (22/34)	PA (25.58 ± 2.83)	
			PA (62.18 ± 14.72)			
Wu et al., 2020 (35)	DAA (39)	Retrospective study	DAA (49.67 ± 9.51)	DAA (15/9)	DAA (22.16 ± 4.17)	15 months
	PA (28)			PA (13/10)	PA (23.32 ± 2.58)	
			PA (48.21 ± 8.64)			
Malek et al., 2016 (36)	DAA (117)	Retrospective study	DAA (70.8)	DAA (117/148)	DAA (28.5)	18.1 months
	PA (183)		PA (70)		PA (29.0)	
				PA (86/97)		
Cao et al., 2020 (37)	DAA (65)	Prospective study	DAA (61.4 ± 12.8)	DAA (27/38)	DAA (24.7 ± 1.9)	1 month
	PA (65)		PA (62.4 ± 8.3)	PA (28/37)	PA (25.1 ± 1.8)	
Dong et al., 2022 (38)	DAA (65)	Retrospective study	DAA (46.0 ± 12.9)	DAA (51/14)	DAA (23.5 ± 3.6)	6 months
	PA (62)		PA (44.7 ± 11.0)	PA (49/13)	PA (23.5 ± 4.0)	
Shen et al., 2023 (39)	DAA (41)	Retrospective study	DAA (59.48 ± 5.14)	DAA (22/19)	DAA (25.18 ± 5.32)	1 year
	PA (335)		PA (65.43 ± 8.51)	PA (157/178)	PA (27.86 ± 4.17)	
Rodriguez et al., 2014 (40)	DAA (60)	Prospective study	N/A	N/A	N/A	1 year
	PA (60)					
Maldonado et al., 2019 (41)	DAA (24)	Retrospective study	DAA（58.9 ± 11.1 ）	DAA (6/18)	DAA (30.9 ± 6.2)	3 months
	PA (24)			PA (6/18)	PA (31.2 ± 5.6)	
			PA (60.1 ± 9.3)			
Moerenhout et al., 2020 (42)	DAA (28)	Prospective study	DAA (70.4 ± 9.1)	DAA (18/9)	DAA (27.6 ± 4.4)	55 months
	PA (27)		PA (68.9 ± 8.8)	PA (11/17)	PA (26.5 ± 4.3)	
Cichos et al., 2023 (43)	DAA (348)	Retrospective study	DAA (70)	DAA (110/238)	DAA (25)	1 year
	PA (197)		PA (68)		PA (26)	
				PA (63/134)		
Kunze et al., 2023 (44)	DAA (20)	Prospective study	DAA (59.47 ± 6.23）	DAA (7/12)	DAA (28.34 ± 5.11)	6 weeks
	PA (20)		PA (62.06 ± 6.25)	PA (7/11)	PA (27.86 ± 5.93)	
Rykov et al., 2017 (45)	DAA (23)	RCT	DAA (62.8 ± 6.1)	DAA (8/15)	DAA (29.0 ± 5.6)	6 weeks
	PA (23)		PA (60.2 ± 8.1)	PA (11/12)	PA (29.3 ± 4.8)	
Maezawa et al., 2022 (46)	DAA (24)	Prospective study	DAA (68.9)	DAA (0/24)	DAA (21.8)	2 weeks
	PA (47)		PA (71.1)	PA (0/47)	PA (23.4)	
Rykov et al., 2021 (47)	DAA (23)	RCT	DAA (62)	DAA (8/15)	DAA (27.8 ± 7.3)	1 year
	PA (23)		PA (63)	PA (11/12)	PA (28.6 ± 8.4)	
De Berardinis et al., 2023 (48)	DAA (92)	Retrospective study	DAA (62.9 ± 7.3)	DAA (54/38)	DAA (25.2 ± 3.1)	10 days
	PA (74)		PA (63.0 ± 6.1)	PA (42/32)	PA (27.3 ± 1.9)	
Chen et al., 2023 (49)	DAA (73)	Retrospective study	DAA (42.82 ± 8.65)	DAA (7/66)	DAA (24.38 ± 3.42)	6 months
	PA (162)			PA (14/148)	PA (24.36 ± 3.45)	
			PA (43.32 ± 9.68)			
Brunello et al., 2023 (50)	DAA (500)	Retrospective study	DAA (64)	DAA (257/243)	N/A	1 year
	PA (147)		PA (66)			
				PA (78/69)		
Jin et al., 2023 (51)	DAA (183)	Retrospective study	DAA (61.1 ± 10.1)	DAA (98/85)	DAA (25.7 ± 4.3)	6 months
	PA (199)		PA (60.8 ± 10.7)	PA (89/110)	PA (25.1 ± 4.5)	
Siljander et al., 2020 (52)	DAA (1,846)	Retrospective study	DAA (65 ± 10)	DAA (844/1,002)	DAA (27.4 ± 4.4)	3 months
			PA (64 ± 11)		PA (30.2 ± 6.2)	
	PA (3,162)			PA (1,363/1,799)		
Lalevée et al., 2022 (53)	DAA (21)	Prospective study	DAA (68. 4 ± 12.5)	DAA (8/13)	DAA (27.5 ± 4.6)	1 year
	PA (21)		PA (71.5 ± 9.2)	PA (8/13)	PA (27 ± 3.7)	
Tsukada and Wakui, 2015 (54)	DAA (139)	Retrospective study	DAA (66.7 ± 9.8)	DAA (14/125)	DAA (23.0 ± 3.0)	DAA (5 years）
	PA (177)		PA (61.7 ± 10.3)	PA (30/147)	PA (23.9 ± 3.7)	
						PA (9 years）
Slaven et al., 2023 (55)	DAA (6,592)	Retrospective study	DAA (62)	N/A	N/A	2.6 years
			PA (64)			
	PA (3,455)					
Charles et al., 2024 (56)	DAA (109)	Retrospective study	DAA (82.3 ± 7.2)	DAA (29/80)	DAA (23.1 ± 5.4)	6 months
			PA (82.6 ± 8.2)	PA (50/121)	PA (23.6 ± 4.5)	
	PA (171)					
Gulbrandsen et al., 2022 (57)	DAA (91)	Retrospective study	DAA (62.7)	DAA (45/46)	DAA (27)	1 year
	PA (90)		PA (59.27)	PA (44/46)	PA (33)	
Yuasa et al., 2018 (58)	DAA (45)	Retrospective study	DAA (63.1 ± 13.1)	DAA (3/39)	DAA (23.9 ± 3.70)	3 months
	PA (43)		PA (60.9 ± 11.7)	PA (5/36)	PA (23.5 ± 3.55)	
Barrett et al., 2013 (59)	DAA (43)	Prospective study	DAA (61.4 ± 9.2)	DAA (29/14)	DAA (30.7 ± 5.4)	1 year
	PA (44)		PA (63.2 ± 7.7)	PA (19/25)	PA (29.1 ± 5.0)	
Wang et al., 2024 (60)	DAA (103)	Retrospective study	DAA (67.3 ± 2.9)	DAA (44/59)	DAA (23.2 ± 1.9)	2 years
	PA (97)		PA (66.6 ± 3.2)	PA (40/57)	PA (22.7 ± 2.0)	
Lan et al., 2022 (61)	DAA (20)	Retrospective study	DAA (52.95 ± 14.76)	DAA (6/14)	DAA (24.09 ± 3.46)	2 years
	PA (22)			PA (4/18)	PA (23.16 ± 4.52)	
			PA (48.91 ± 16.84)			
Chung et al., 2022 (62)	DAA (36)	Retrospective study	DAA (78.19 ± 9.44)	DAA (3/33)	DAA (22.60 ± 4.26)	2 days
	PA (31)					
				PA (5/26)	PA (22.18 ± 5.81)	
			PA (76.45 ± 6.79)			
Liu et al., 2021 (63)	DAA (23)	Retrospective study	DAA (42.0 ± 13.6)	DAA (4/18)	DAA (21.9 ± 3.2)	DAA (28.5 months)
	PA (50)		PA (47.8 ± 17.6)	PA (14/33)	PA (23.5 ± 4.3)	
						PA (39.0 months)
Wang et al., 2022 (64)	DAA (47)	Retrospective study	DAA (65.2 ± 4.4)	DAA (25/22)	DAA (23.5 ± 1.1)	1 year
	PA (33)		PA (64.7 ± 5.2)	PA (19/14)	PA (23.2 ± 1.4)	
Yang et al., 2021 (65)	DAA (20)	RCT	DAA (49.4 ± 13.3)	DAA (15/5)	DAA (24.1 ± 2.6)	1 year
	PA (20)		PA (49.4 ± 13.3)	PA (15/5)	PA (24.1 ± 2.6)	

BMI, body mass index; DAA, direct anterior approach; PA, posterior approach; RCT, randomized controlled trial; SD, standard deviation.

### Assessment of ROB in the included studies

**Figure 2:**
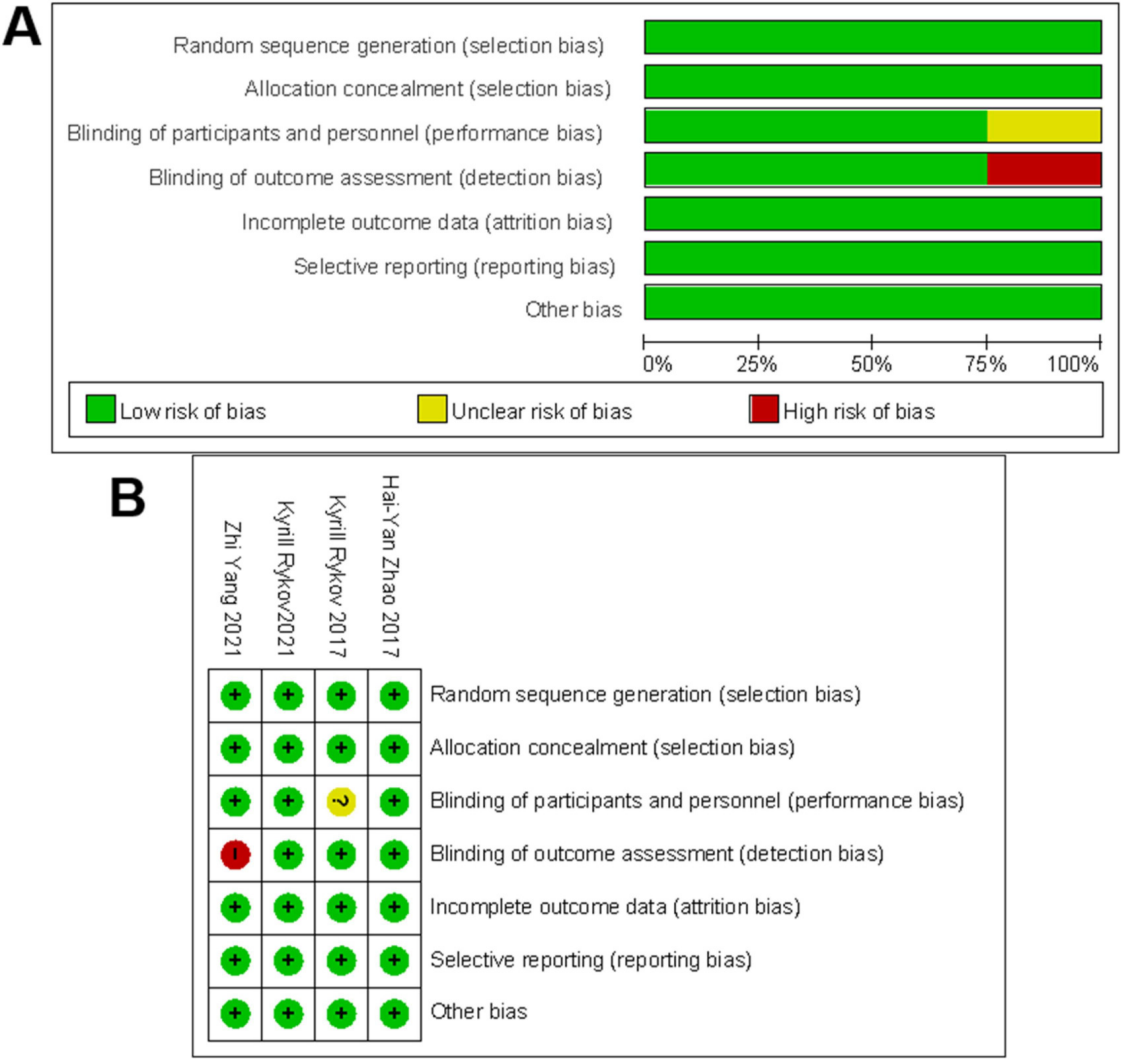
The methodological quality assessment for RCTs. **(A)** Risk-of-bias graph for included studies. **(B)** Risk-of-bias summary for included studies. +, no bias; −, bias; ?, bias unknown.

The quality of the included RCTs and case–control studies was evaluated by the Cochrane Collaboration tool, NOS, and MINORS. As shown in Figure 2, all four RCTs were assessed as high quality. Additionally, 44 case–control studies scored at least eight points on the NOS, suggesting relatively stable methodological quality (Tables 2, 3).

**Table 2:** Quality assessment of case–control studies.

Study	Is the case definition adequate?	Representativeness of the cases	Selection of controls	Definition of controls	Comparability of cases and controls based on the design or analysis	Ascertainment of exposure	Same method of ascertainment for cases and controls	Non-response rate	Total
Di Martino et al., 2023 (18)	1	1	1	1	1	1	1	1	8
Alexander Martusiewicz 2020 (19)	1	1	1	1	1	1	1	1	8
Spaans et al., 2012 (20)	1	1	1	1	1	1	1	1	8
Bremer et al., 2011 (21)	1	1	1	1	1	1	1	1	8
Fransen et al., 2016 (22)	1	1	1	1	1	1	1	1	8
Zhang et al., 2022 (23)	1	1	1	1	1	1	1	1	8
Chen et al., 2020 (24)	1	1	1	1	2	1	1	1	9
Yin et al.,2024 (25)	1	1	1	1	1	1	1	1	8
Agten et al., 2017 (26)	1	1	1	1	1	1	1	1	8
Ponzio et al., 2018 (27)	1	1	1	1	2	1	1	1	9
Maldonado et al., 2019 (28)	1	1	1	1	2	1	1	1	9
Rhea et al., 2020 (29)	1	1	1	1	1	1	1	1	8
Pala et al., 2016 (30)	1	1	1	1	1	1	1	1	8
Vasarhelyi et al., 2020 (31)	1	1	1	1	1	1	1	1	8
Triantafyllopoulos et al., 2019 (32)	1	1	1	1	1	1	1	1	8
Sprowls et al., 2020 (33)	1	1	1	1	2	1	1	1	9
Wu et al., 2020 (35)	1	1	1	1	1	1	1	1	8
Malek et al., 2016 (36)	1	1	1	1	1	1	1	1	8
Cao et al., 2020 (37)	1	1	1	1	1	1	1	1	8
Dong et al., 2022 (38)	1	1	1	1	1	1	1	1	8
Shen et al., 2023 (39)	1	1	1	1	2	1	1	1	9
Rodriguez et al., 2014 (40)	1	1	1	1	1	1	1	1	8
Maldonado et al., 2019 (41)	1	1	1	1	1	1	1	1	8
Moerenhout et al., 2020 (42)	1	1	1	1	1	1	1	1	8
Cichos et al., 2023 (43)	1	1	1	1	2	1	1	1	9
Kunze et al., 2023 (44)	1	1	1	1	1	1	1	1	8
Maezawa et al., 2022 (46)	1	1	1	1	1	1	1	1	8
De Berardinis et al., 2023 (48)	1	1	1	1	2	1	1	1	9
Chen et al., 2023 (49)	1	1	1	1	1	1	1	1	8
Brunello et al., 2023 (50)	1	1	1	1	1	1	1	1	8
Jin et al., 2023 (51)	1	1	1	1	1	1	1	1	8
Siljander et al., 2020 (52)	1	1	1	1	1	1	1	1	8
Lalevée et al., 2022 (53)	1	1	1	1	1	1	1	1	8
Tsukada and Wakui, 2015 (54)	1	1	1	1	1	1	1	1	8
Slaven et al., 2023 (55)	1	1	1	1	1	1	1	1	8
Charles et al., 2024 (56)	1	1	1	1	1	1	1	1	8
Gulbrandsen et al., 2022 (57)	1	1	1	1	1	1	1	1	8
Yuasa et al., 2018 (58)	1	1	1	1	1	1	1	1	8
Barrett et al., 2013 (59)	1	1	1	1	1	1	1	1	8
Wang et al., 2024 (60)	1	1	1	1	1	1	1	1	8
Lan et al., 2022 (61)	1	1	1	1	1	1	1	1	8
Chung et al., 2022 (62)	1	1	1	1	1	1	1	1	8
Liu et al., 2021 (63)	1	1	1	1	1	1	1	1	8
Wang et al., 2022 (64)	1	1	1	1	1	1	1	1	8

**Table 3:** Methodological index for non-randomized studies (MINORS).

Study	A clearly stated aim	Inclusion of consecutive patients	Prospective collection of data	Endpoints appropriate to the aim of the study	Unbiased assessment of the study endpoint	Follow-up period appropriate to the aim of the study	Loss to follow-up <5%	Prospective calculation of the study size	Total
Di Martino et al., 2023 (18)	2	1	2	2	2	1	2	2	14
Alexander Martusiewicz 2020 (19)	2	2	1	2	2	1	2	2	15
Spaans et al., 2012 (20)	2	2	2	2	2	2	2	2	16
Bremer et al., 2011 (21)	2	2	1	2	2	2	2	1	15
Fransen et al., 2016 (22)	2	2	1	2	2	2	2	2	15
Zhang et al., 2022 (23)	2	2	0	2	2	2	2	2	14
Chen et al., 2020 (24)	2	2	2	2	2	2	2	2	16
Yin et al.,2024 (25)	2	2	2	2	2	2	2	2	16
Agten et al., 2017 (26)	2	2	2	2	2	2	2	2	16
Ponzio et al., 2018 (27)	2	2	2	2	2	2	2	2	16
Maldonado et al., 2019 (28)	2	2	2	2	2	2	2	2	16
Rhea et al., 2020 (29)	2	2	2	2	2	2	0	2	14
Pala et al., 2016 (30)	2	2	2	2	2	2	2	2	16
Vasarhelyi et al., 2020 (31)	2	2	2	2	2	2	2	2	16
Triantafyllopoulos et al., 2019 (32)	2	2	2	2	2	2	2	2	16
Sprowls et al., 2020 (33)	2	2	2	2	2	2	2	2	16
Wu et al., 2020 (35)	2	2	1	2	2	2	2	2	15
Malek et al., 2016 (36)	2	2	2	2	2	2	2	2	16
Cao et al., 2020 (37)	2	2	2	2	2	2	2	2	16
Dong et al., 2022 (38)	2	2	2	2	2	2	2	2	16
Shen et al., 2023 (39)	2	2	2	2	2	2	2	2	16
Rodriguez et al., 2014 (40)	2	2	2	2	2	2	2	2	16
Maldonado et al., 2019 (41)	2	2	2	2	2	2	2	2	16
Moerenhout et al., 2020 (42)	2	2	2	2	2	2	2	2	16
Cichos et al., 2023 (43)	2	2	2	2	2	2	2	2	16
Kunze et al., 2023 (44)	2	2	2	2	2	2	2	2	16
Maezawa et al., 2022 (46)	2	2	2	2	2	1	2	2	15
Berardinis et al., 2023 (48)	2	2	2	2	2	1	2	2	15
Chen et al., 2023 (49)	2	2	2	2	2	2	2	2	16
Brunello et al., 2023 (50)	2	2	2	2	2	2	2	2	16
Jin et al., 2023 (51)	2	2	2	2	2	2	2	2	16
Siljander et al., 2020 (52)	2	2	2	2	2	2	2	2	16
Lalevée et al., 2022 (53)	2	2	2	2	2	2	2	2	16
Tsukada and Wakui, 2015 (54)	2	2	2	2	2	2	2	2	16
Slaven et al., 2023 (55)	2	2	2	2	2	2	2	2	16
Charles et al., 2024 (56)	2	2	2	2	2	2	2	2	16
Gulbrandsen et al., 2022 (57)	2	2	2	2	2	2	2	2	16
Yuasa et al., 2018 (58)	2	2	2	2	2	2	2	2	16
Barrett et al., 2013 (59)	2	2	2	2	2	2	2	2	16
Wang et al., 2024 (60)	2	2	2	2	2	2	2	2	16
Lan et al., 2022 (61)	2	2	2	2	2	2	2	2	16
Chung et al., 2022 (62)	2	2	2	2	2	2	2	2	16
Liu et al., 2021 (63)	2	2	2	2	2	2	2	2	16
Wang et al., 2022 (64)	2	2	2	2	2	2	2	2	16

### Surgical trauma

#### Blood transfusion rate

**Figure 3:**
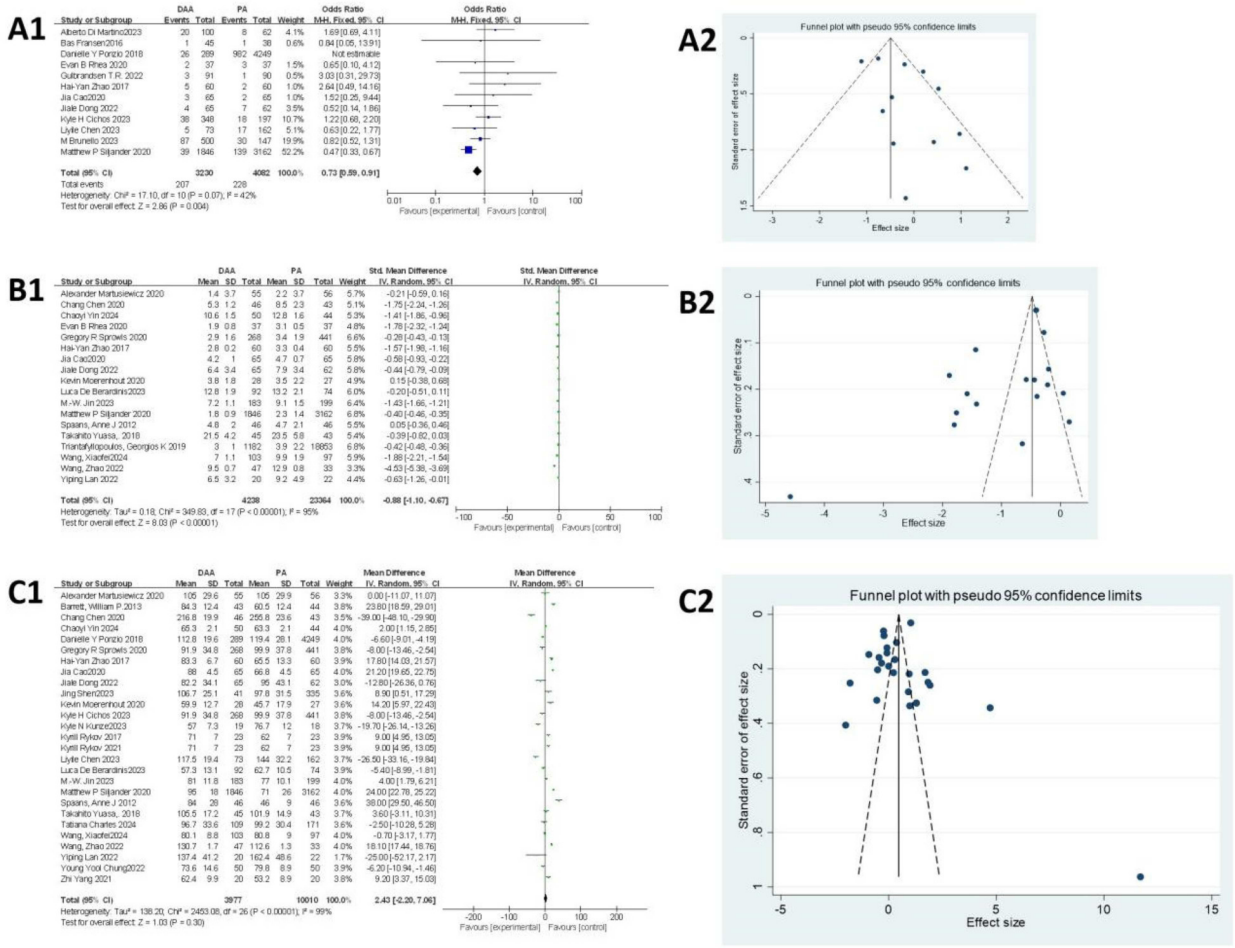
Comparison between DAA and PA in the surgical trauma-related subject for **(A1, A2)** blood transfusion rate, **(B1, B2)** hospital stay, and **(C1, C2)** surgery time. DAA, direct anterior approach; PA, posterior approach; Fixed, fixed-effects model; Random, random-effects model; M–H, Mantel–Haenszel; CI, confidence intervals; MD, mean difference.

As shown in Figure 3A1, 12 articles (18, 22, 27, 29, 34, 37, 38, 43, 49, 50, 52, 57) evaluated the blood transfusion rate. Egger's test (Figure 3A2) indicated no publication bias (*P* > 0.05). The initial fixed-effects model revealed heterogeneity (*I^2^ *= 62%, *P* = 0.21). The study by Ponzio et al. (27) was identified as the primary source of heterogeneity due to significant sample size imbalance between groups and was excluded from further analysis. After exclusion, the fixed-effects model showed a significantly lower blood transfusion rate in the DAA group compared with that in the PA group [6.62% vs. 14.52%; *I *^2 ^= 42%, OR = 0.73, 95% confidence interval (CI): 0.59–0.91, *P* < 0.005].

#### Hospital stay

As shown in Figure 3B1, 18 articles (19, 20, 24, 25, 29, 32–34, 37, 38, 42, 48, 51, 52, 58, 60, 61, 64) assessed hospital stay. Egger's test (Figure 3B2) revealed significant publication bias (*P* < 0.05), primarily attributed to the studies by Chen et al. (24), Rhea et al. (29), Zhao et al. (34), Jin et al. (51), Wang et al. (60), and Wang et al. (64). After excluding these seven studies, heterogeneity was significantly reduced, allowing the use of a fixed-effects model. The results indicated that hospital stay was significantly shorter in the DAA group than that in the PA group (*I*^2^ = 36%, 95% CI: −0.43 to −0.36, *P* < 0.001).

#### Surgery time

As shown in Figure 3C1, surgery time was reported in 27 studies (19, 20, 24, 25, 27, 33, 34, 37–39, 42–45, 47–49, 51, 52, 56, 58–62, 64, 65), involving 13,987 hips (3,977 in the DAA group and 10,010 in the PA group). Egger's test (Figure 3C2) indicated no publication bias (*P* > 0.05). Due to significant heterogeneity in the fixed-effects model, subgroup analysis based on body mass index (BMI) was performed; however, this did not substantially reduce heterogeneity (Attachment 1). A random-effects model was therefore applied, revealing no significant difference in surgery time between the DAA and PA groups (*I*^2^ = 99%, 95% CI: −2.20 to 7.06, *P* = 0.30).

### Muscle damage

#### MRI findings

**Figure 4:**
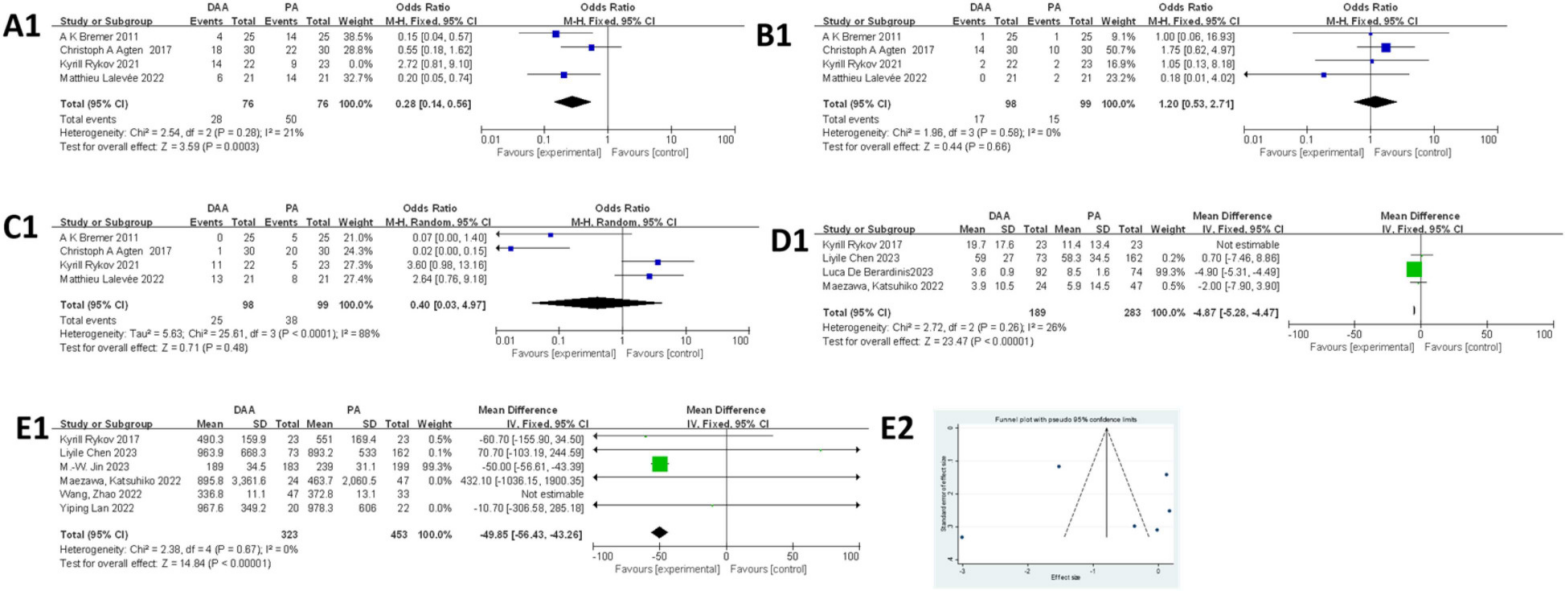
Comparison between DAA and PA in muscle damage-related factor for **(A1)** the glutes minimus damage on MRI, **(B1)** the gluteus medius damage on MRI, **(C1)** the tensor fasciae latae damage on MRI, and **(D1)** CRP and **(E1, E2)** CK levels. DAA, direct anterior approach; PA, posterior approach; Fixed, fixed-effects model; Random, random-effects model; M–H, Mantel–Haenszel; CI, confidence intervals; MD, mean difference.

Muscle damage around the hip joint was assessed using the Goutallier score (66), where a score of >2 was considered to indicate muscle damage according to previous reports (47). As shown in Figures 4A1–C1, four studies (21, 26, 45, 53) assessed muscle damage using MRI, including damage to the gluteus minimus, gluteus medius, and tensor fasciae latae muscles. For the gluteus minimus muscle damage, the fixed-effects model initially showed significant heterogeneity (*I *^2 ^= 76%, *P* = 0.02). After excluding the study by Rykov K, which was identified as the primary source of heterogeneity, the heterogeneity was reduced (*I*^2^ = 21%, *P* < 0.05). The final analysis showed that the DAA resulted in less muscle damage compared with the PA (36.84% vs. 65.79%, OR = 0.28; 95% CI: 0.14−0.56; *P* < 0.005). For the gluteus medius muscle damage, the fixed-effects model indicated no significant difference between the DAA and PA groups (OR =  1.20; 95% CI: 0.53–2.71; *I*^2^ = 0%, *P* = 0.66). For the tensor fasciae latae muscle damage, substantial heterogeneity was observed in the fixed-effects model (*I *^2 ^= 88%, *P* = 0.07). Therefore, a random-effects model was applied. The analysis showed no significant difference between the two approaches (OR = 0.40; 95% CI: 0.03−4.97; *I*^2^ = 88%, *P* = 0.48). Due to the small sample sizes, Egger's test could not be used to assess publication bias.

#### CRP levels

As shown in Figure 4D1, four studies (45, 46, 48, 49) were included in the CRP level analysis. The fixed-effects model revealed significant heterogeneity (*I*^2^ = 72%, *P* < 0.00001). After excluding the study by Rykov et al., which was identified as the primary source of heterogeneity, the heterogeneity was substantially reduced (*I*^2^ = 26%, *P* < 0.00001). The final analysis, involving 472 hips (189 in the DAA group and 283 in the PA group), showed that CRP levels were significantly lower in the DAA group compared with those in the PA group [mean difference (MD) = −4.87; 95% CI: −5.28 to −4.47; *P* < 0.00001]. Due to the small sample size, Egger's test could not be used to assess publication bias.

#### CK levels

As shown in Figure 4E1, six studies (45, 46, 49, 51, 61, 64) were included in the meta-analysis of CK levels. Egger’s test (Figure 4E2) showed no evidence of publication bias (*P* > 0.05). The initial fixed-effects model indicated significant heterogeneity (*I*^2^ = 60%, *P* < 0.00001). After excluding the study by Wang et al. (64), identified as the main source of heterogeneity, the heterogeneity was eliminated (*I*^2^ = 0%, *P* < 0.00001). The final results showed that CK levels were lower in the DAA group compared with those in the PA group.

### Complications

#### Infection

**Figure 5:**
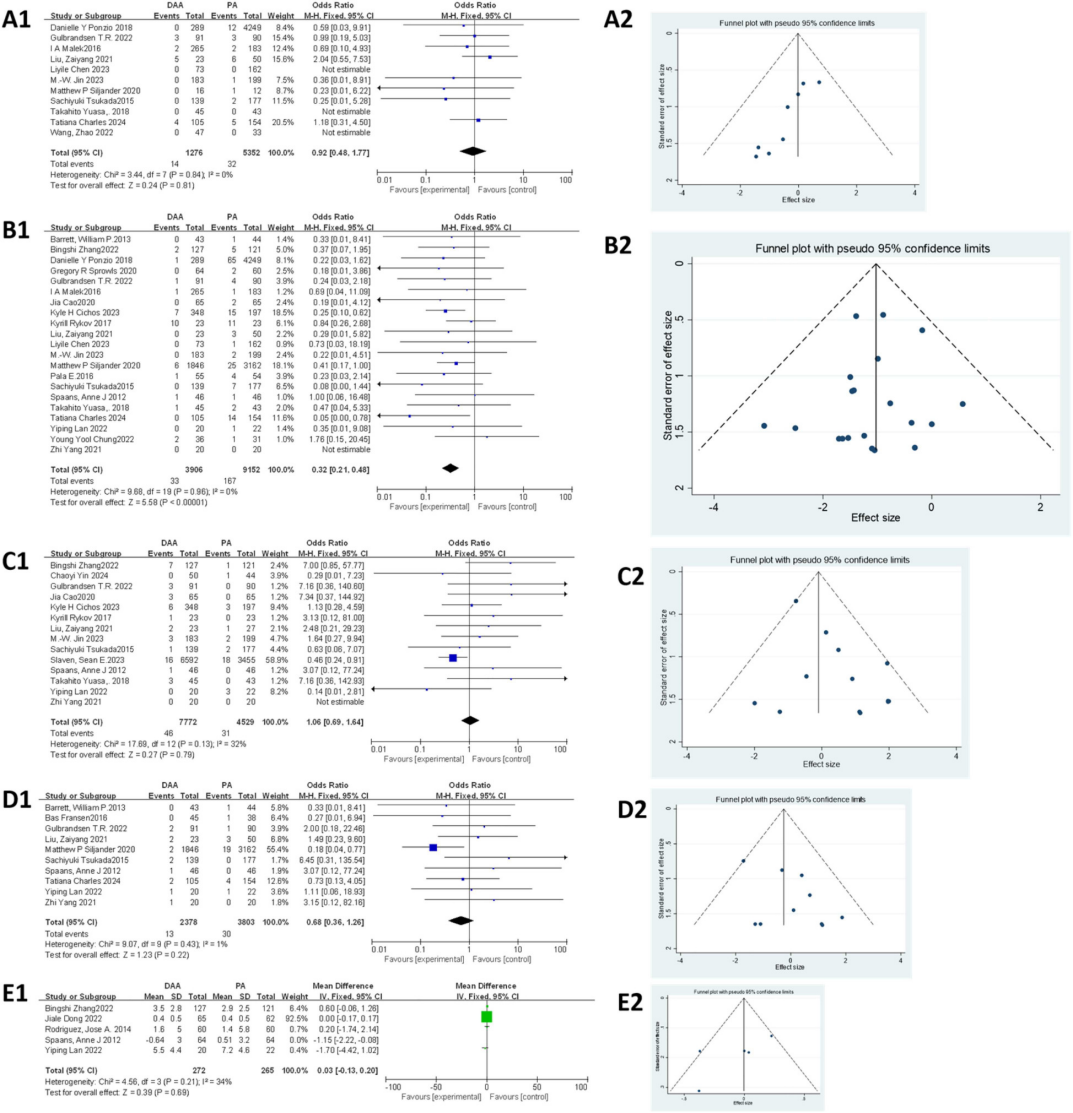
Comparison between DAA and PA in complications-related factor for **(A1, A2)** infection rate, **(B1, B2)** dislocation rate, **(C1, C2)** nerve injury rate, **(D1, D2)** intraoperative fracture rate, and **(E1, E2)** posterior leg length discrepancy. DAA, direct anterior approach; PA, posterior approach; Fixed, fixed-effects model; Random, random-effects model; M–H, Mantel–Haenszel; CI, confidence intervals; MD, mean difference.

As shown in Figure 5A1, 11 studies (27, 36, 49, 51, 52, 54, 56–58, 63, 64) involving 6,628 hips (1,276 in the DAA group and 5,352 in the PA group) were included in the infection rate analysis. Egger's test (Figure 5A2) indicated publication bias (*P* < 0.05). In addition, the fixed-effects model showed low heterogeneity (*I*^2^ = 0%, *P* = 0.81), and there was no significant difference in infection rates between the two groups (OR = 0.92; 95% CI: 0.48–1.77; *P* = 0.81).

#### Dislocation

As shown in Figure 5B1, 21 studies (20, 23, 27, 30, 33, 36, 37, 43, 45, 49, 51, 52, 54, 56–59, 61–63, 65) involving 13,058 hips (3,902 in the DAA group and 9,856 in the PA group) were analyzed for dislocation rates. Egger's test (Figure 5B2) showed no publication bias (*P* > 0.05). The fixed-effects model (*I*^2^ = 0%, *P* < 0.001) revealed that the dislocation rate was significantly lower in the DAA group compared with that in the PA group (0.84% vs. 1.82%; 95% CI: 0.20–0.48; *P*  <  0.00001; *I*^2^ = 0%).

#### Nerve injury

As shown in Figure 5C1, 14 studies (20, 23, 25, 37, 43, 45, 51, 54, 55, 57, 58, 61, 63, 65) involving 12,301 hips (7,772 in the DAA group and 4,529 in the PA group) were included in the nerve injury analysis. Egger's test (Figure 5C2) indicated publication bias (*P* < 0.05). In addition, the fixed-effects model showed low heterogeneity (*I*^2^ = 32%, *P* = 0.79), and the analysis revealed no significant difference in nerve injury between the two groups (95% CI: 0.69–1.64; *P* = 0.79).

#### Intraoperative fracture

As shown in Figure 5D1, 10 studies (20, 22, 52, 54, 56, 57, 59–61, 65) involving 6,181 hips (2,378 in the DAA group and 3,803 in the PA group) were included in the meta-analysis. Egger's test (Figure 5D2) indicated no publication bias (*P* > 0.05). In addition, the fixed-effects model showed low heterogeneity (*I*^2^ = 1%, *P* = 0.22), and the results demonstrated no significant difference in intraoperative fracture rates between the DAA and PA groups (95% CI: 0.36–1.26; *P* = 0.22).

#### Leg length discrepancy

As shown in Figure 5E1, five studies (20, 23, 38, 40, 61) involving 665 hips (336 in the DAA group and 329 in the PA group) were included in the meta-analysis. Egger's test (Figure 5E2) indicated no publication bias (*P* > 0.05). In addition, the fixed-effects model initially showed moderate heterogeneity (*I*^2^ = 56%, *P* = 0.95), which was significantly reduced after excluding the study by Spaans et al. (20) (*I*^2^ = 34%, *P* = 0.69). The final results showed no significant difference in postoperative leg length discrepancy between the DAA and PA groups (95% CI: −0.13 to 0.20; *P* = 0.69).

### Function scores

#### HHS

**Figure 6:**
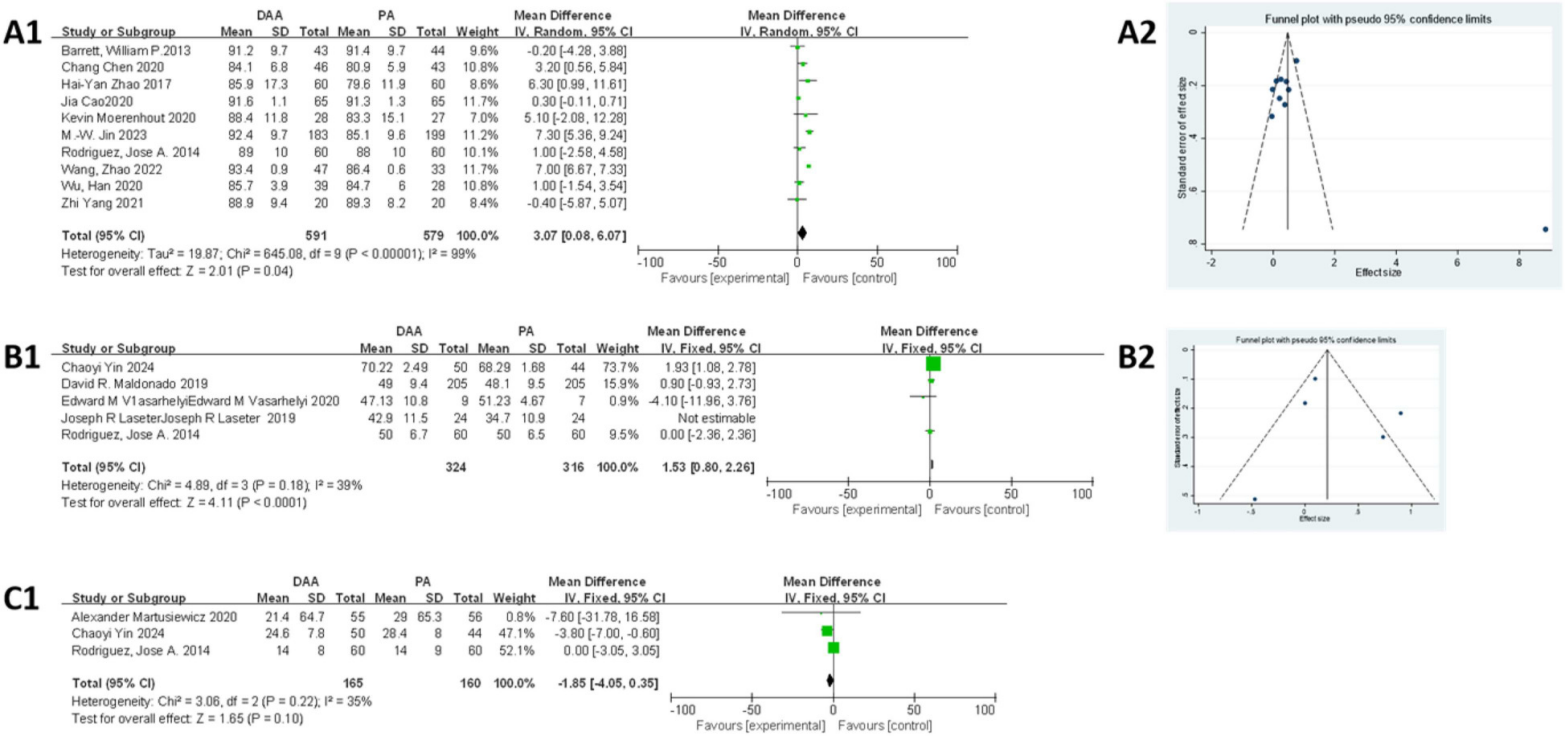
Comparison between DAA and PA in function score for **(A1, A2)** postoperative HHS score, **(B1, B2)** postoperative SF score, **(C1)** time to discontinuation of a walker after surgery. DAA, direct anterior approach; PA, posterior approach; Fixed, fixed-effects model; Random, random-effects model; M–H, Mantel–Haenszel; CI, confidence intervals; MD, mean difference.

As shown in Figure 6A1, 10 studies (23, 34, 35, 37, 40, 42, 51, 59, 64, 65) were eligible for this meta-analysis. Egger's test (Figure 6A2) indicated no publication bias (*P* > 0.05). Due to significant heterogeneity in the fixed-effects model (*I*^2^ = 99%, *P* < 0.00001), subgroup analysis based on BMI was performed but did not reduce heterogeneity (Attachment 2). A random-effects model was applied, and the results indicated that the DAA group had significantly higher HHS compared with that in the PA group (MD = 3.07, 95% CI: 0.08–6.07; *I*^2^ = 99%, *P* < 0.05).

#### SF score

As shown in Figure 6B1, five studies (25, 28, 31, 40, 41) were included in the meta-analysis. Egger's test (Figure 6B2) indicated no publication bias (*P* > 0.05). However, the fixed-effects model showed moderate heterogeneity (*I*^2^ = 56%, *P* < 0.00001), which was clearly reduced after excluding the study by Maldonado et al. (41) (*I*^2^ = 39%, *P* < 0.00001). The final results showed that patients in the DAA group had significantly higher SF scores compared with those in the PA group (MD = 1.53, 95% CI: 0.80–2.26; *P* < 0.01).

#### Time to discontinuation of a walker after surgery

As shown in Figure 6C1, three studies (19, 25, 40) were included in this meta-analysis. The fixed-effects model demonstrated low heterogeneity (*I^2^ =* 35%), and the results showed no significant difference between the DAA and PA groups in terms of time to discontinuation of a walker postoperatively (MD = −1.85, 95% CI: −4.05 to 0.35; *P* = 0.10). Due to the small sample size, Egger's test could not be used to assess publication bias.

## Discussion

This meta-analysis compared ERAS-related outcomes between the DAA and PA in THA. The results showed that the DAA group had a lower blood transfusion rate, shorter hospital stay, reduced gluteus minimus muscle damage on MRI, lower postoperative levels of CK and CRP, and significantly higher HHS and SF scores. Additionally, the DAA group had a lower dislocation rate compared with that in the PA group. However, no significant differences were found in surgery time, damage to the gluteus medius and tensor fasciae latae muscles on MRI, time to discontinuation of a walker after surgery, or complication rates (infection, nerve injury, intraoperative fracture, and postoperative leg length discrepancy).

ERAS can reduce surgical trauma, minimize postoperative complications, and promote faster recovery (67). Muscle damage indicators such as MRI-based Goutallier score and serum CK (68) and CRP (60) levels have been widely used to assess perioperative soft tissue injury (59, 63, 68). In the present analysis, the DAA demonstrated less gluteus minimus muscle damage and lower postoperative CK and CRP levels, highlighting its minimally invasive feature. No significant differences were observed in gluteus medius or tensor fasciae latae muscle damage, which may be attributed to the fact that both approaches have minimal involvement with these muscles. Although previous studies suggested that the DAA caused greater damage to the tensor fasciae latae and required longer surgery time (5), more recent evidence suggests that surgeons' experience can significantly reduce this impact, especially after overcoming the learning curve (69). In addition, variations in healthcare systems, rehabilitation protocols, and patient populations may contribute to discrepancies in hospitalization duration. Collectively, these findings indicate that the DAA, with less muscle injury, is associated with a lower blood transfusion rate and reduced length of hospital stay.

This meta-analysis evaluated the rate of complications, including infection, dislocation, nerve injury, intraoperative fracture, and postoperative leg length discrepancy, in the DAA and PA groups. Among these, the DAA showed a significant advantage only in the dislocation rate. While some previous studies reported a higher rate of femoral fractures with the DAA, our meta-analysis found no significant difference between the two groups. It could be related to surgeons' proficiency and the occurrence of minor proximal femoral cleavages that do not compromise the stability of the prosthesis, which also commonly occur within the PA. Regarding nerve injury, the impact of both approaches on nerve damage was inconsistent. It is generally believed that the DAA approach is more likely to cause lateral femoral cutaneous nerve injury, while the PA approach is more associated with sciatic nerve injury, resulting in no significant difference in nerve injury rates between the two approaches. Additionally, it was previously thought that the DAA provided better control over leg length due to its performance in the supine position (67, 70). However, with the widespread adoption of the DAA in the lateral decubitus position, this advantage has diminished (71). Therefore, the transition from the supine to the lateral decubitus position may account for the loss of DAA's advantage in leg length control.

Functional outcomes, such as the HHS, SF score, and time to discontinuation of a walker after surgery, were closely related to surgical trauma, muscle damage, and postoperative complications. The DAA, with its lower dislocation rate and reduced muscle damage, resulted in better functional scores, especially in pain relief and mental improvement. The DAA group also had significantly higher SF scores compared with those in the PA group, which is likely due to less muscle damage, lower dislocation rates, and shorter hospital stay. However, these significant differences may still not necessarily translate into meaningful real-world benefits for patients.

Several limitations should be considered in this meta-analysis. (1) Only four RCTs were included, resulting in a relatively low level of evidence. (2) Methodological inconsistencies across the included studies led to data deviations that may have affected the findings. (3) The analysis did not account for surgeons' proficiency due to the absence of a learning curve evaluation. Learning curves are an important factor, as previous studies (72) have shown that even experienced surgeons can experience a significant increase in surgical time and postoperative complications during the early stages of learning. Unfortunately, studies comparing learning curves between the DAA and PA were lacking and therefore not included in this analysis. (4) High heterogeneity in the measurement of some outcomes may have affected the results, potentially due to publication bias and selection bias. Future research should adopt stricter inclusion criteria and standardized outcome reporting to reduce heterogeneity and improve the reliability of findings. (5) The two approaches were not the same in terms of muscle injury. The DAA was performed between the tensor fasciae latae and sartorius, making significant damage to the gluteus medius and minimus very unlikely. Similarly, the PA passes through the gluteus maximus, meaning damage to the gluteus medius and minimus was also not expected. So the future studies should focus on assessing potential damage to the gluteus maximus and tensor fasciae latae.

## Conclusion

Based on the results of our meta-analysis, the DAA demonstrated the advantages of minimally invasive surgery, including less muscle damage, fewer postoperative complications, and better functional outcomes compared with the PA in the context of ERAS protocols. Therefore, we recommend that surgeons consider adopting the DAA in ERAS protocols, provided that the patient meets the surgical criteria for this approach.
